# Prevalence of Obesity and Its Impact on Outcome in Patients With COVID-19: A Systematic Review and Meta-Analysis

**DOI:** 10.3389/fendo.2021.598249

**Published:** 2021-02-25

**Authors:** Nafiye Helvaci, Nesrin Damla Eyupoglu, Erdem Karabulut, Bulent Okan Yildiz

**Affiliations:** ^1^ Division of Endocrinology and Metabolism, Department of Internal Medicine, Hitit University School of Medicine, Corum, Turkey; ^2^ Division of Endocrinology and Metabolism, Department of Internal Medicine, Hacettepe University School of Medicine, Ankara, Turkey; ^3^ Department of Biostatistics, School of Medicine, Hacettepe University, Ankara, Turkey

**Keywords:** obesity, body mass index, COVID-19, SARS-CoV-2, prognosis

## Abstract

**Background and Objective:**

Obesity has been reported as a risk factor for adverse outcomes in COVID-19. However, available studies presenting data on obesity prevalence in patients with COVID-19 have conflicting results. The objective of this systematic review and meta-analysis is to evaluate the prevalence of obesity in these patients and to stratify the estimates by illness severity.

**Methods:**

We performed a literature search with the use of Medline/PubMed and Google Scholar database from December 1, 2019 to June 27, 2020 and systematically reviewed studies reporting the number of obese patients with real-time reverse transcriptase polymerase chain reaction (rRT-PCR)-confirmed SARS-CoV-2 infection.

**Results:**

Nineteen studies were identified. The pooled obesity prevalence rates were 0.32 (95% CI: 0.24–0.41) in hospitalized patients, 0.41 (95% CI: 0.36–0.45) in patients admitted to intensive care unit, 0.43 (95% CI: 0.36–0.51) in patients needing invasive mechanic ventilation (IMV), and 0.33 (95% CI: 0.26–0.41) in those who died. Obesity was associated with a higher risk for hospitalization [Odds ratio (OR): 1.3, 95% CI: 1.00–1.69; I^2^ 52%, *p* = 0.05], ICU admission (OR: 1.51, 95% CI: 1.16–1.97; I^2^ 72%, *p* = 0.002), and IMV requirement (OR: 1.77, 95% CI: 1.34–2.35; I^2^ 0%, *p* < 0.001). The increase in risk of death did not reach statistical significance (OR: 1.28, 95% CI: 0.76–2.16, p = 0.35) which might be due to obesity survival paradox and/or unidentified factors.

**Conclusions:**

Our data indicate that obese subjects may be at higher risk for serious illness if infected and obesity may play a role in the progression of COVID-19.

## Introduction

Coronavirus disease 19 (COVID-19) caused by severe acute respiratory syndrome coronavirus 2 (SARS-CoV-2) has rapidly spread and become a global pandemic, with more than 13 million confirmed cases resulting in over 585,000 confirmed deaths as of July 17, 2020 (1). Clinical manifestations of COVID-19 vary in a broad spectrum, ranging from asymptomatic or mild infection to life-threatening acute respiratory distress syndrome and multiorgan failure. Older age and the presence of comorbidities including hypertension (HT), type 2 diabetes (T2DM), and cardiovascular disease (CVD) seems to be associated with a more severe course of COVID-19 (2–4).

Obesity represents a major and urgent global health problem (5). It tends to increase with increasing age and is a known risk factor for the abovementioned comorbidities identified as predisposing factors for adverse outcomes in COVID-19 (5). Although there are several reports evaluating the burden of obesity on the clinical course of COVID-19, it has not been fully documented whether people living with obesity have a higher risk of getting COVID-19. Most of the earlier studies on COVID-19 did not provide information about body mass index (BMI) or other measures of adiposity of the patients (2–4, 6–8). Other studies which present data on obesity prevalence in patients with COVID-19 have conflicting results, reporting similar, lower, or higher rates of obesity compared to general population (9–12). The aim of this systematic review and meta-analysis was, therefore, to evaluate the prevalence of obesity in patients with COVID-19 and to stratify the estimates by illness severity.

## Material and Methods

### Protocol and Registration

We report this systematic review and meta-analysis in accordance with the Preferred Reporting Items for Systematic Reviews and Meta-Analyses (PRISMA) Statement (13). The review protocol was registered on the International Prospective Register of Systematic Reviews (PROSPERO) database (CRD42020199145).

### Literature Search Strategy

The following medical subject titles, key words, and their combinations were used to search on Medline/PubMed and Google Scholar database for retrospective cohorts, cross-sectional and longitudinal studies including gray literature as pre-prints, conference papers, and reports which were accessed online between December 1, 2019 and June 27, 2020: 2019 nCoV, SARS-CoV-2, COVID-19, coronavirus disease 2019, obesity, body mass index, BMI, clinical features, risk factors. Reference lists of relevant articles were also screened to capture other potentially eligible studies. The literature search was concluded by June 27, 2020 and only reports written in English language were assessed.

### Eligibility Criteria and Study Selection

The primary outcome measure was to evaluate the overall prevalence of obesity in COVID-19 infection and stratify the estimates by disease severity and geographic region. Studies reporting the number of obese patients with real-time reverse transcriptase polymerase chain reaction (rRT-PCR)-confirmed SARS-CoV-2 infection at least in two of the following groups were included in the meta-analysis: all cases, hospitalized patients, patients admitted to an intensive care unit (ICU), patients needing invasive mechanical ventilation (IMV), and patients who died. Duplicate publications, reviews, perspectives and letters not presenting original data, studies lacking information on BMI or obesity were excluded, as well as studies including any intervention, pediatric population, patients with any specific condition (e.g., malnutrition, pregnancy), or fewer than 50 patients. Among studies involving the same patient groups, the largest cohort was selected. Studies that applied a BMI cut-off value other than ≥30 kg/m^2^ for obesity were also excluded; however, studies defining obesity as BMI ≥28 kg/m^2^ in Asian Pacific populations were eligible. To avoid selection bias, two reviewers (NH and ND) identified and selected articles that met the inclusion criteria. The full text of each study was assessed independently by these reviewers and any disagreement among them was discussed and resolved by consensus or discussion with a third reviewer (BY).

### Data Extraction and Quality Assessment

Information on first author, publication year, country, study design, time and duration of follow-up, total sample size, gender composition, obesity criteria, numbers reported for obesity at different outcome levels were extracted from all included studies. The methodological quality of studies was evaluated through the National Heart, Lung, and Blood Institute Quality Assessment Tool for Observational Cohort and Cross-Sectional Studies (14). Data extraction and quality assessment was conducted independently by two reviewers (NH and NE). Disagreements were resolved by joint discussion or by the third reviewer (BY).

### Statistical Analyses

All statistical analyses were done through metaprop and metabin commands in meta package version 4.13 in R software ver. 3.6.3. Meta-analysis of obesity prevalence was performed through the DerSimonian-Laird, random-effects method, to account for a high heterogeneity with 95% confidence interval. The Freeman–Tukey double arcsine transformation was applied for prevalences to normalize and stabilize the variance of the sampling distribution. Higgins’ I² was used to assess heterogeneity with significance set at >75%.

## Results

### Literature Search

**Figure 1:**
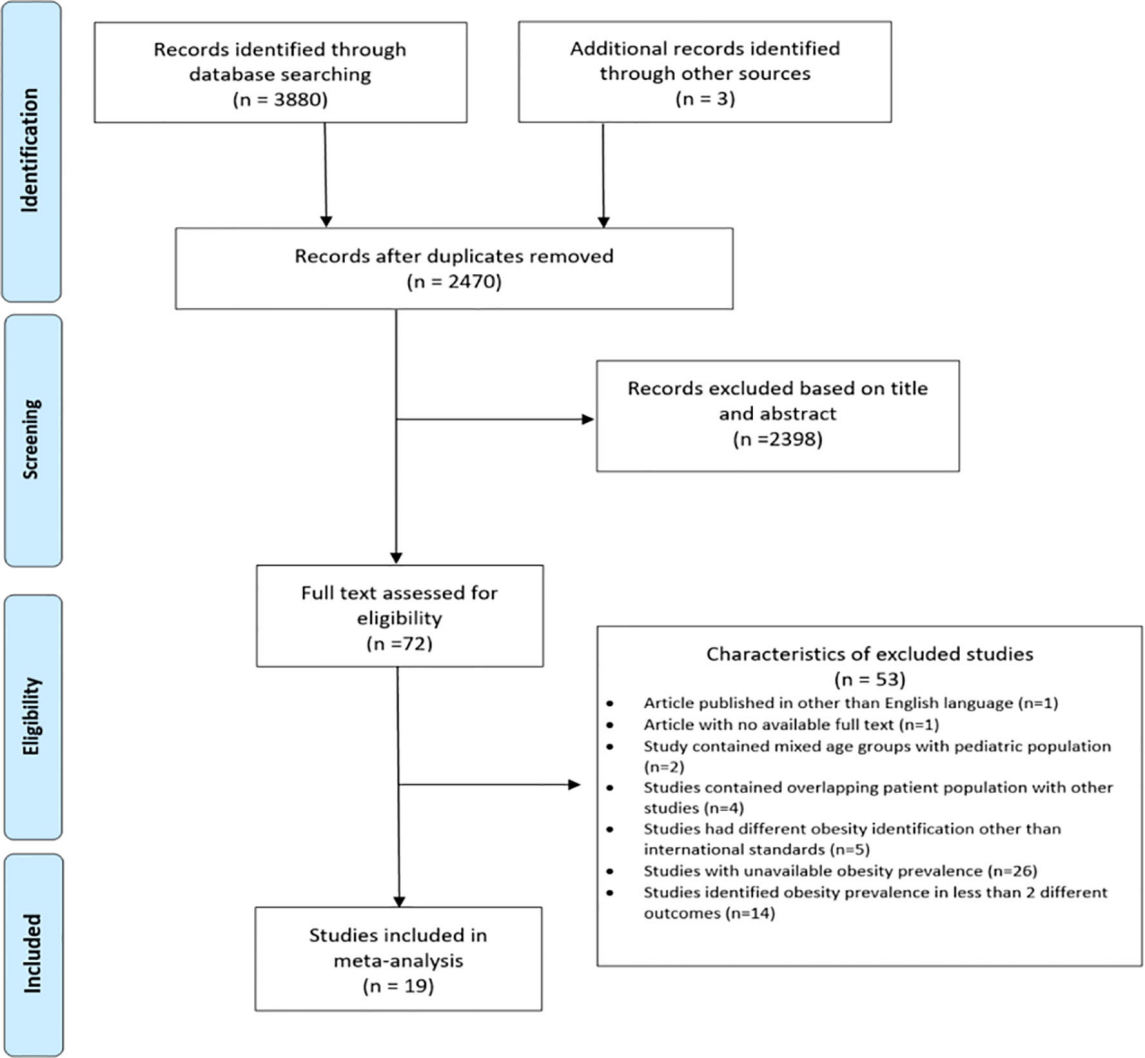
PRISMA study flow diagram of the search and selection process.

Initial search strategy identified a total of 3,883 records, of which 2,470 remained after the removal of duplicates. After assessment of titles and abstracts of these records, 72 articles were found to be potentially relevant. Full texts of these articles were reviewed and finally, 19 studies (9–12, 15–29) that met the inclusion criteria were included in this systematic review and meta-analysis. A PRISMA study flow diagram of this search and selection process is shown in **Figure 1**.

### Study Characteristics

The main characteristics of the included studies are summarized in **Table 1**. The results of the risk of bias assessment of individual studies are shown in **Table 2**. All included studies were observational cohort studies. Their sample size ranged from 50 to 51,633, describing a total of 68,214 patients having confirmed COVID-19. The research time period of the included studies ranged from January 11 to May 18, 2020. Out of 19 studies, eight were conducted in the U.S., two in Mexico, six in Europe, two in China, and one in Kuwait respectively.

**Table 1:** Characteristics of the included studies.

Author	Country	Design	Period	Sample size/male (%)	Age*	Ethnicity	Obesity, n (%)	Missing BMI data (%)	Outcome
**North America**									
Argenziano et al.	U.S.	Retrospective	March 1–April 5, 2020	1,000/59.6	63 (50–75)	White 18%, Hispanic 31%, Black 22%, Asian 2%, other 27%	352 (41.9)	15.9	Hospitalization, ICU admission, IMV, mortality
Ebinger et al.	U.S.	Retrospective	March 8–21,2020	442/57.9	52.7 ± 19.7	White 69%, Black 14%, Asian 8%, other 9%	71 (16)	0	Clinical illness severity
Hajifathalian et al.	U.S.	Retrospective	March 4–April 9, 2020	770/60.8	63.5 ± 16.7	White 41%, Black 14%, Asian 16%, other 29%	277 (36)	0	ICU admission, IMV, mortality
Hur et al.	U.S.	Retrospective	March 1–April 8, 2020	486/55.8	59 (19–101)	White 39%, Hispanic 23%, Black 23%, Asian 4%, other 4%	259 (53.3)	0	IMV
Kaligeros et al.	U.S.	Retrospective	February 17–April 5, 2020	103/61.2	60 (50–72)	White 41%, Hispanic 23%, Black 34%, Asian 2%,	49 (47.5)	0	ICU admission, IMV
Petrilli et al.	U.S.	Prospective	March 1–April 8, 2020	5,279/49.5	54 (38–66)	White 40%, Hispanic 27%, Black 17%, Asian 8%, other 8%	1865 (35.3)	4.5	Hospitalization, critical illness, mortality
Pettit et al.	U.S.	Prospective	March 1–April 18, 2020	238/47.5	58.5 ± 17	White 5%, Hispanic 1%, Black 91%, Asian 1%, other 3%	146 (61.3)	0	Length of stay, IMV, ICU admission, mortality
Suleyman e al.	U.S.	Retrospective	March 9–27, 2020	463/44.1	57.5 ± 16.8	Black 72%, other 28%	262 (57.6)	0	Hospitalization, ICU admission, IMV
Bello-Chavolla et al.	Mexico	Retrospective	Until May 18, 2020	51,633/57.7	46.7 ± 15.8	NR	10708 (20.7)	0	Hospitalization, ICU admission, IMV, mortality
Ortiz-Brizuela et al.	Mexico	Prospective	February 26–April 11, 2020	309/60.7	43 (33–54)	NR	67 (39.6)	45.3	Hospitalization, ICU admission
**Asia**									
Al-Sabah et al.	Kuwait	Retrospective	February 24–April 7, 2020	1,158/81.6	40.5 (32–52)	Indian 47.5%, Kuwaiti 26%, other 26.5	148 (20.4)	37.2	ICU admission
Cai et al.	China	Retrospective	January 11–February 21, 2020	383/47.8	Range 28–62	NR	41 (10.7)	0	Clinical illness severity, mortality
Hu et al.	China	Retrospective	January 8–February 20, 2020	323/51.4	61 (23–91)	NR	13 (4)	0	Clinical illness severity, unfavorable outcome
**Europe**									
Caussy et al.	France	Retrospective	February 27–April 8, 2020	291/NR	NR	NR	96 (33)	0	IMV
Dreher et al.	Germany	Retrospective	February–March 2020	50/66	65 (58–76)	NR	17 (34)	0	ARDS, ICU admission, mortality
Giacomelli et al.	Italy	Prospective	February 21–March 19, 2020	233/69.1	61 (50–72)	NR	38 (16.3)	0	Mortality
ICNARC	UK	Registry-based cohort	March 1–April 19, 2020	6720/71.8	60 (52–68)	White 72%, Black 12%, Asian 17%, other 9%	2310 (38.5)	10.6	Hospitalization, ICU admission, IMV, mortality
Rottoli et al.	Italy	Retrospective	February 28–March 28, 2020	296/65.2	Obese 68.3 ± 13.5, non-obese 63 ± 16.8	NR	70 (23.6)	0	Hospitalization, discharge, respiratory failure, ICU admission, mortality
Simonnet et al.	France	Retrospective	February 27–April 5, 2020	124/73	60 (51-70)	NR	59 (47.6)	0	IMV

ARDS, acute respiratory distress syndrome; BMI, body mass index; ICNARC, Intensive Care & National Audit Research Center; ICU, intensive care unit; IMV, invasive mechanical ventilation; NR, not reported; UK, United Kingdom; U.S., United States.

*Data are reported as median (interquartile range) or mean ± standard deviation.

**Table 2:** Quality assessment of enrolled studies.

Study	Question number^a^														Quality
	1	2	3	4	5	6	7	8	9	10	11	12	13	14	
Argenziano et al.	Yes	Yes	Yes	No	No	Yes	Yes	No	Yes	No	Yes	No	NA	No	Good
Ebinger et al.	Yes	Yes	CD	No	No	Yes	No	No	Yes	No	Yes	No	NA	Yes	Fair
Hajifathalian et al.	Yes	Yes	Yes	No	No	Yes	Yes	No	Yes	No	Yes	No	NA	Yes	Good
Hur et al.	Yes	Yes	Yes	No	No	Yes	Yes	Yes	Yes	No	Yes	No	NA	Yes	Good
Kaligeros et al.	Yes	Yes	CD	No	No	Yes	Yes	Yes	Yes	No	Yes	No	NA	Yes	Good
Petrilli et al.	Yes	Yes	CD	No	No	Yes	Yes	Yes	Yes	No	Yes	No	CD	Yes	Good
Pettit et al.	Yes	Yes	CD	No	No	Yes	Yes	Yes	Yes	No	Yes	No	NA	Yes	Good
Suleyman et al.	Yes	Yes	CD	No	No	Yes	No	Yes	Yes	No	Yes	No	NA	Yes	Good
Ortiz-Brizuela et al.	Yes	Yes	Yes	CD	No	Yes	Yes	No	Yes	No	Yes	No	NA	No	Good
Bello-Chavolla et al.	Yes	Yes	Yes	CD	No	Yes	CD	No	Yes	No	Yes	No	CD	Yes	Good
Al-Sabah et al.	Yes	Yes	Yes	No	No	Yes	Yes	Yes	Yes	No	Yes	No	NA	Yes	Good
Hu et al.	Yes	Yes	CD	CD	No	Yes	Yes	No	Yes	No	No	No	NA	No	Fair
Cai et al.	Yes	Yes	CD	CD	No	Yes	Yes	No	Yes	No	Yes	No	NA	Yes	Good
Caussy et al.	Yes	Yes	CD	CD	No	Yes	Yes	Yes	Yes	No	Yes	No	NA	No	Good
Dreher et al.	Yes	Yes	CD	CD	No	Yes	CD	No	Yes	No	Yes	No	NA	No	Fair
Giacomelli et al.	Yes	Yes	CD	CD	No	Yes	No	No	Yes	No	Yes	No	CD	Yes	Fair
ICNARC	No	Yes	Yes	No	No	Yes	Yes	No	Yes	No	Yes	No	CD	No	Fair
Rottoli et al.	Yes	Yes	CD	CD	No	Yes	Yes	No	Yes	No	Yes	No	NA	Yes	Good
Simonnet et al.	Yes	Yes	CD	CD	No	Yes	Yes	Yes	Yes	No	Yes	No	NA	No	Good

a The questions are those of the National Heart, Lung, and Blood Institute Quality Assessment Tool for Observational Cohort and Cross-Sectional Studies (14). #1. Was the research question or objective in this paper clearly stated? #2. Was the study population clearly specified and defined? #3. Was the participation rate of eligible persons at least 50%? #4. Were all the subjects selected or recruited from the same or similar populations (including the same time period)? Were inclusion and exclusion criteria for being in the study prespecified and applied uniformly to all participants? #5. Was a sample size justification, power description, or variance and effect estimates provided? #6. For the analyses in this paper, were the exposure(s) of interest measured prior to the outcome(s) being measured? #7. Was the timeframe sufficient so that one could reasonably expect to see an association between exposure and outcome if it existed? #8. For exposures that can vary in amount or level, did the study examine different levels of the exposure as related to the outcome (e.g., categories of exposure, or exposure measured as continuous variable)? #9. Were the exposure measures (independent variables) clearly defined, valid, reliable, and implemented consistently across all study participants? #10. Was the exposure(s) assessed more than once over time? #11. Were the outcome measures (dependent variables) clearly defined, valid, reliable, and implemented consistently across all study participants? #12. Were the outcome assessors blinded to the exposure status of participants? #13. Was loss to follow-up after baseline 20% or less? #14. Were key potential confounding variables measured and adjusted statistically for their impact on the relationship between exposure(s) and outcome(s)?

CD, cannot determine; NA, not applicable.

Five studies with 58,419 patients provided data for the combined analysis (inpatient + outpatient), 15 studies with 7,758 patients reported on hospitalization, 17 studies with 10,391 patients reported on ICU admission, and 9 studies with 5,107 patients reported on IMV requirement.

Anthropometric measurements were performed at hospital admission in only two studies (16, 22). In the rest, data from previous medical records were used to define obesity. Out of 19 studies, five had missing BMI data of the patients (9, 11, 20, 21, 29). The proportion of patients without BMI data ranged from 4.5 to 45.3% in these studies.

### Prevalence of Obesity in Patients With COVID-19

**Figure 2:**
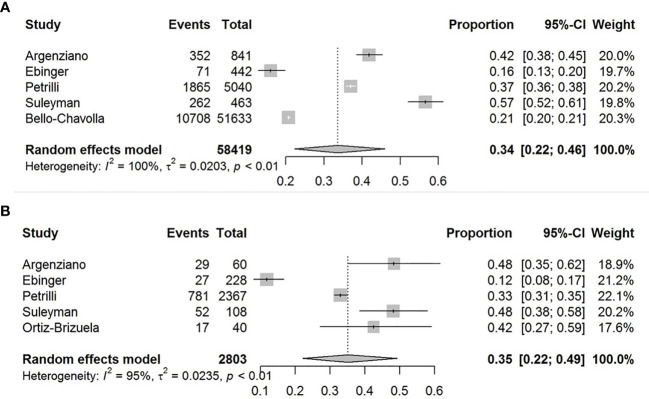
Forest-plots showing the prevalence rates of obesity in all cases **(A)** and in non-admitted patients with COVID-19 **(B)**.

The pooled obesity prevalence rate in all cases (admitted + non-admitted) with COVID-19 was 0.34 [95% confidence interval (CI): 0.22–0.46] as presented in **Figure 2A**. The pooled obesity prevalence rate was 0.35 (95% CI: 0.22–0.49) in non-admitted patients (**Figure 2B**). There was significant heterogeneity among studies. I^2^ estimates were 100 and 95%, respectively (p < 0.01 for both).

**Figure 3:**
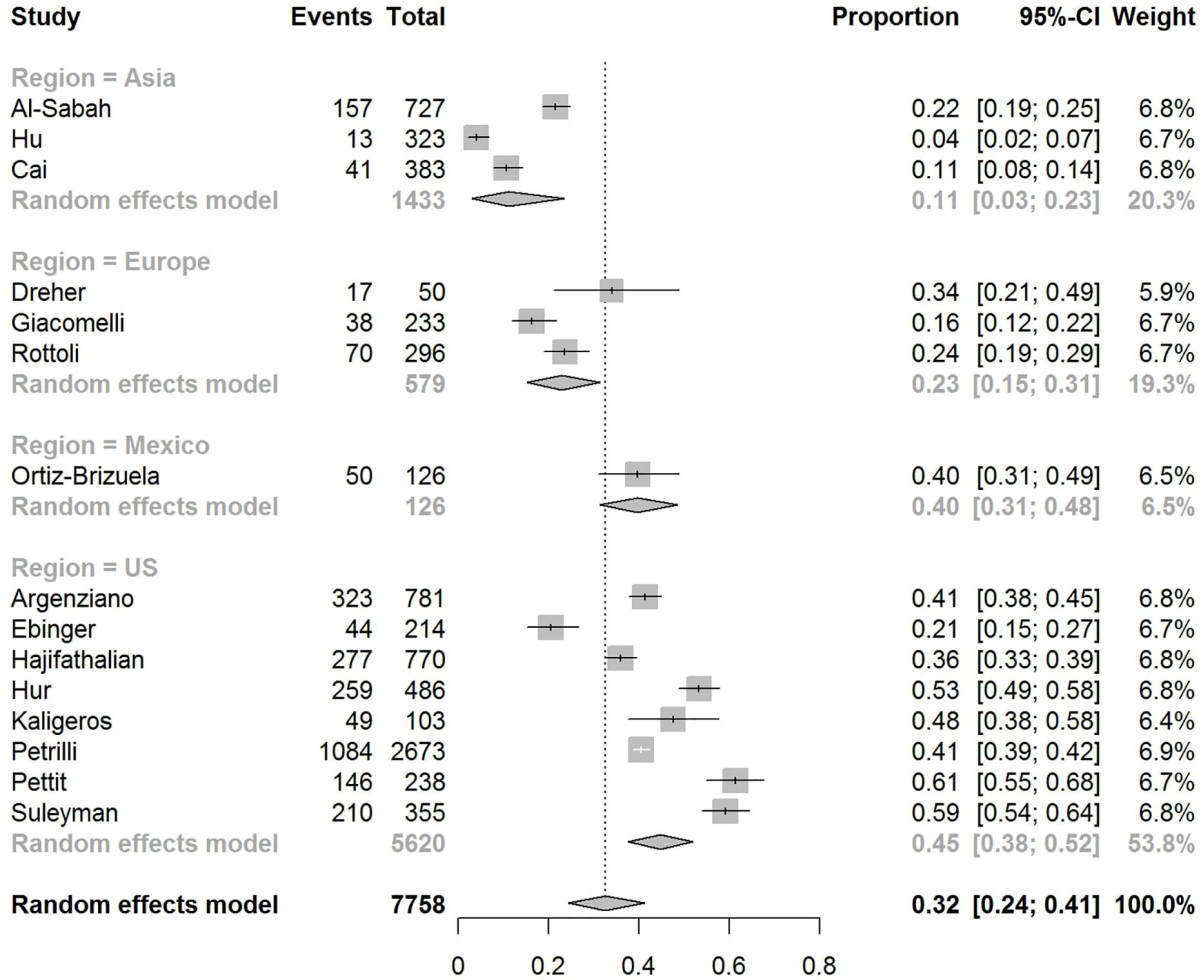
Forest-plot showing the prevalence rate of obesity in hospitalized patients with COVID-19.

**Figure 4:**
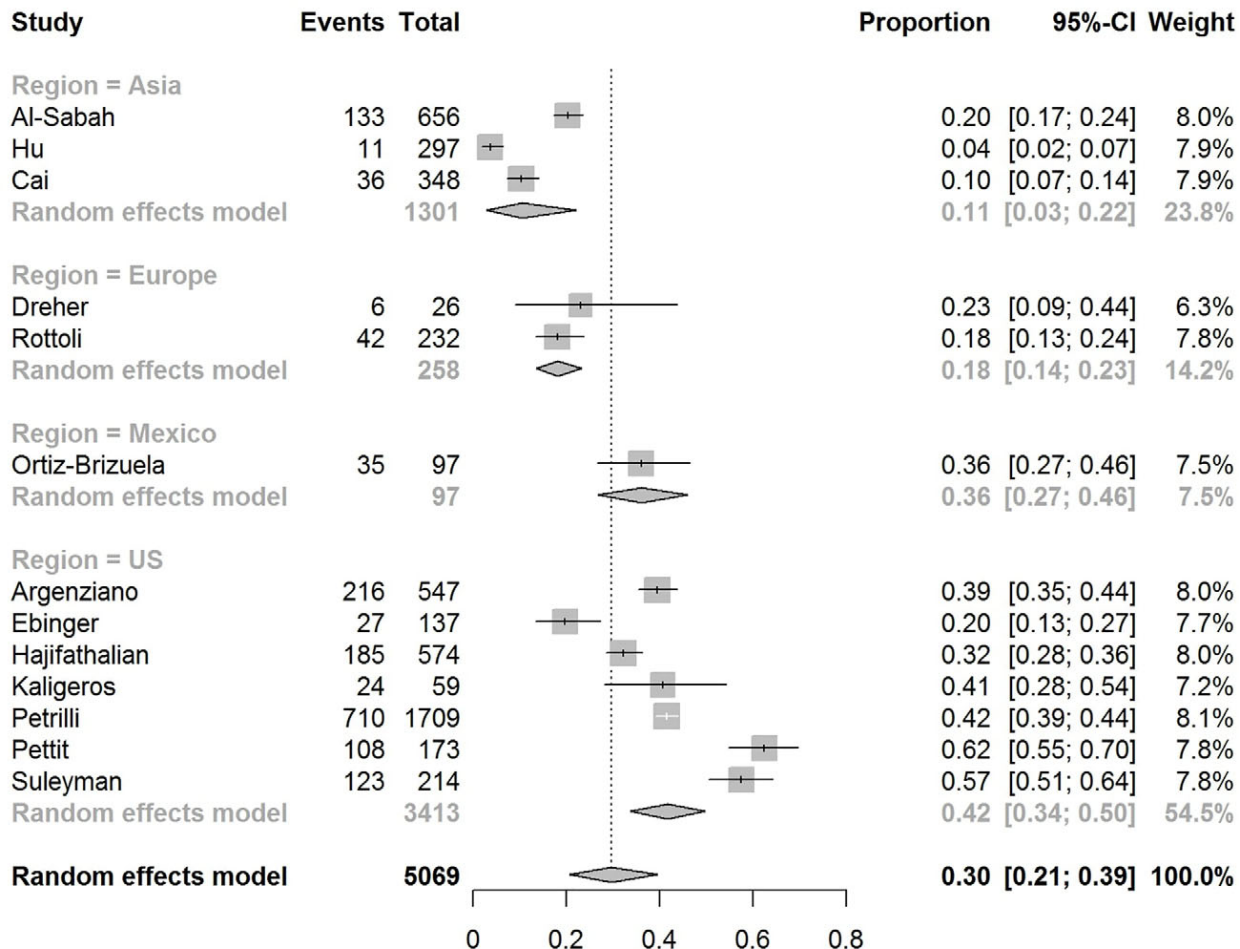
Forest-plot showing the prevalence rate of obesity in patients with COVID-19 admitted to non-critical care wards.

**Figure 5:**
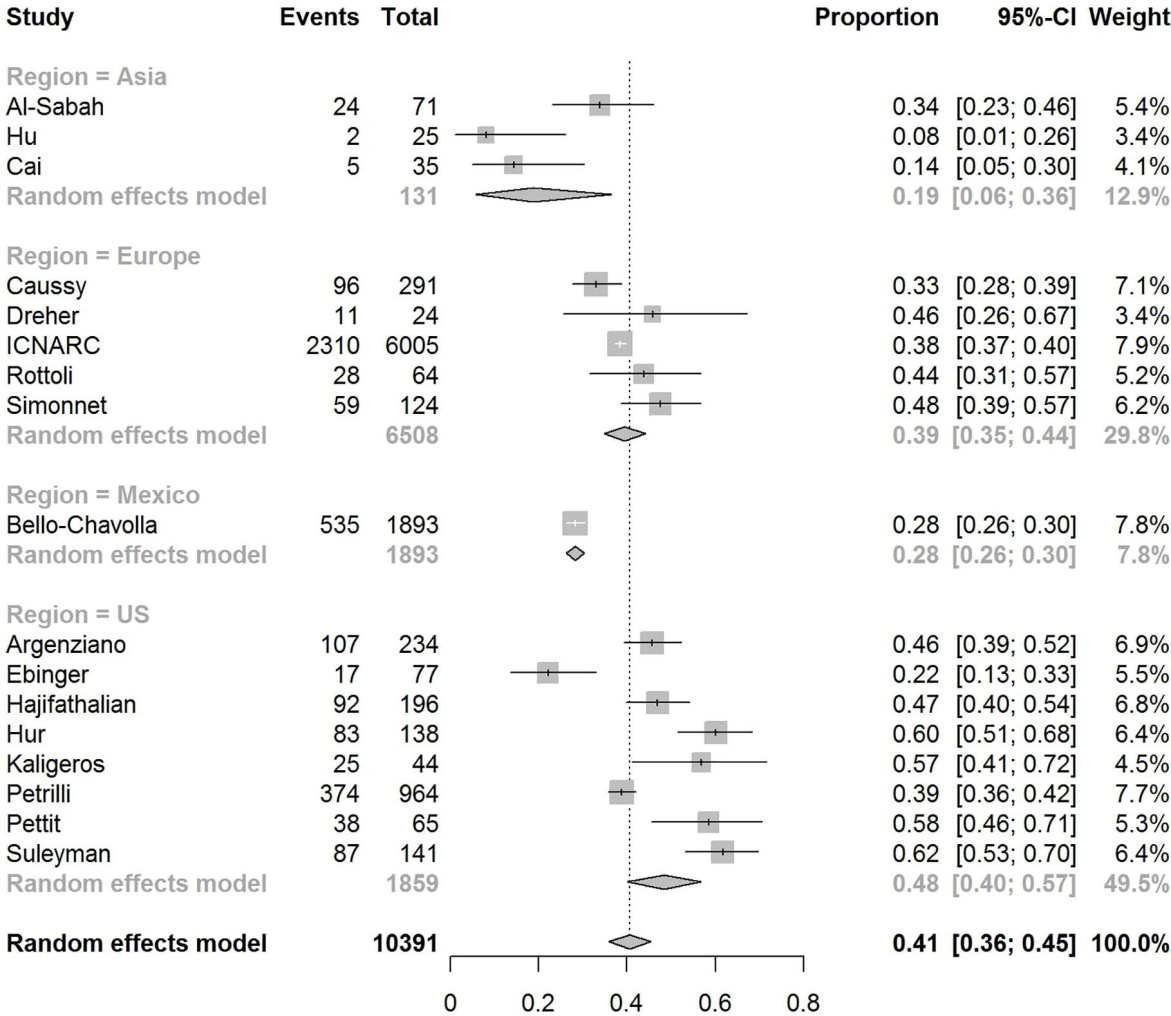
Forest-plot showing the prevalence rate of obesity in patients with CVOID-19 admitted to intensive care unit (ICU).

**Figure 6:**
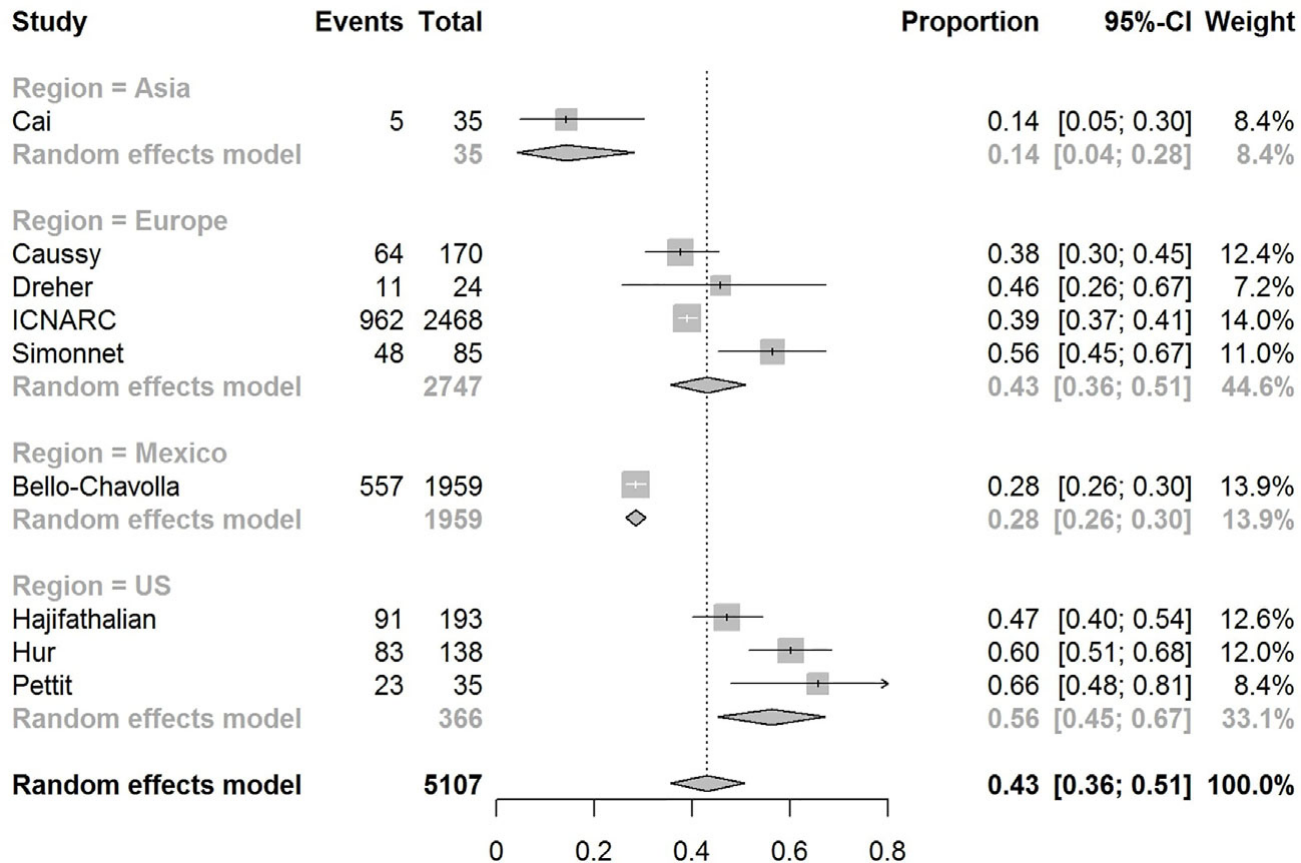
Forest-plot showing the prevalence rate of obesity in patients with COVID-19 needing invasive mechanical ventilation (IMV).

**Figure 7:**
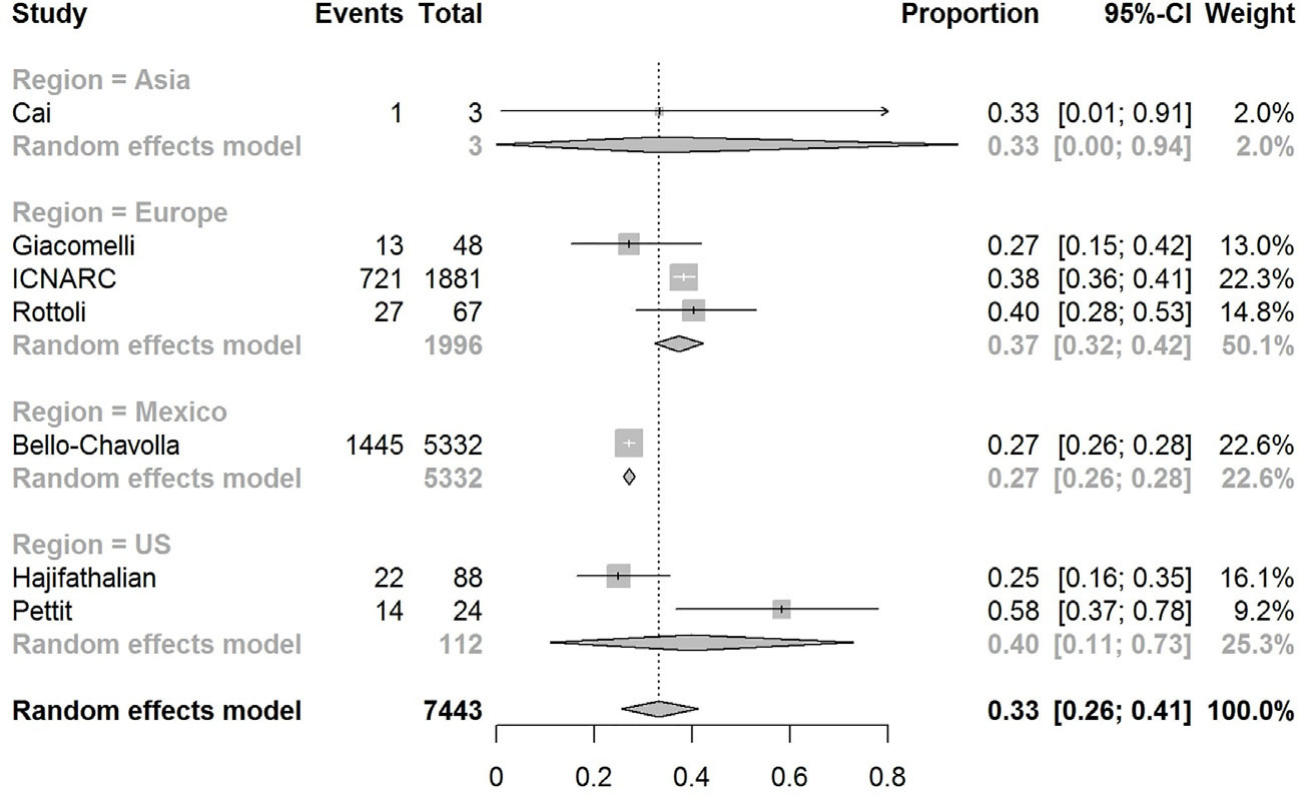
Forest-plot showing the prevalence rate of obesity in deceased patients with COVID-19.

The pooled obesity prevalence rates were 0.32 (95% CI: 0.24–0.41) in hospitalized patients (**Figure 3**), 0.30 (95% CI: 0.21–0.39) in patients admitted to non-critical care wards (**Figure 4**), 0.41 (95% CI: 0.36–0.45) in patients admitted to an ICU (**Figure 5**), 0.43 (95% CI: 0.36–0.51) in patients with IMV requirement (**Figure 6**), and 0.33 (95% CI: 0.26–0.41) in those who died (**Figure 7**) respectively. Studies from the U.S., Europe, and Asia were included in these analyses. All rates were significantly higher than the 13.2% worldwide prevalence of obesity (30). I^2^ estimates were 98, 98, 92, 94, and 94%, indicating significant heterogeneity among studies (all p < 0.01).

Subgroup analysis according to the geographic region were as follows:

In the U.S., the prevalence rates of obesity were 0.45 (95% CI: 0.38–0.52) in hospitalized patients (**Figure 3**), 0.42 (95% CI: 0.34–0.50) in patients admitted to non-critical care wards (**Figure 4**), 0.48 (95% CI: 0.40–0.57) in patients admitted to ICU (**Figure 5**), 0.56 (95% CI: 0.45–0.67) in patients with IMV requirement (**Figure 6**), and 0.40 (95% CI: 0.11–0.73) in those who died (**Figure 7**) respectively. Obesity rate was higher only in patients who required IMV compared to the background general population prevalence of obesity in the U.S., which is 42.4% (31).

In Europe, the prevalence rates of obesity were 0.23 (95% CI: 0.15–0.31) in hospitalized patients (**Figure 3**), 0.18 (95% CI: 0.14–0.23) in patients admitted to non-critical care wards (**Figure 4**), 0.39 (95% CI: 0.35–0.44) in patients admitted to ICU (**Figure 5**), 0.43 (95% CI: 0.36–0.51) in patients with IMV requirement (**Figure 6**), and 0.37 (95% CI: 0.32–0.42) in those who died (**Figure 7**) respectively. Prevalence rates of obesity in patients admitted to ICU, in patients needing IMV, and in those who died were significantly higher than the obesity prevalence of the countries where the included studies were conducted, which are 27.8% in UK, 22.3% in Germany, 21.6% in France, and 19.9% in Italy (32).

In Asia, the prevalence rates of obesity were 0.11 (95% CI: 0.03–0.23) in hospitalized patients (**Figure 3**), 0.11 (95% CI: 0.03–0.22) in patients admitted to non-critical care wards (**Figure 4**), 0.19 (95% CI: 0.06–0.36) in patients admitted to ICU (**Figure 5**), 0.14 (95% CI: 0.04–0.28) in patients with IMV requirement (**Figure 6**), and 0.33 (95% CI: 0.00–0.94) in those who died (**Figure 7**) respectively. Among the included studies, one was conducted in Kuwait (approximately 48% of included patients were Indian), two in China. Compared to the general obesity prevalences, which are 6.2% in China, 3.9% in India, and 37.9% in Kuwait (32), these rates are not significantly increased. However, the wide confidence intervals should be considered while interpreting these results.

### Association Between Obesity and Disease Severity

**Figure 8:**
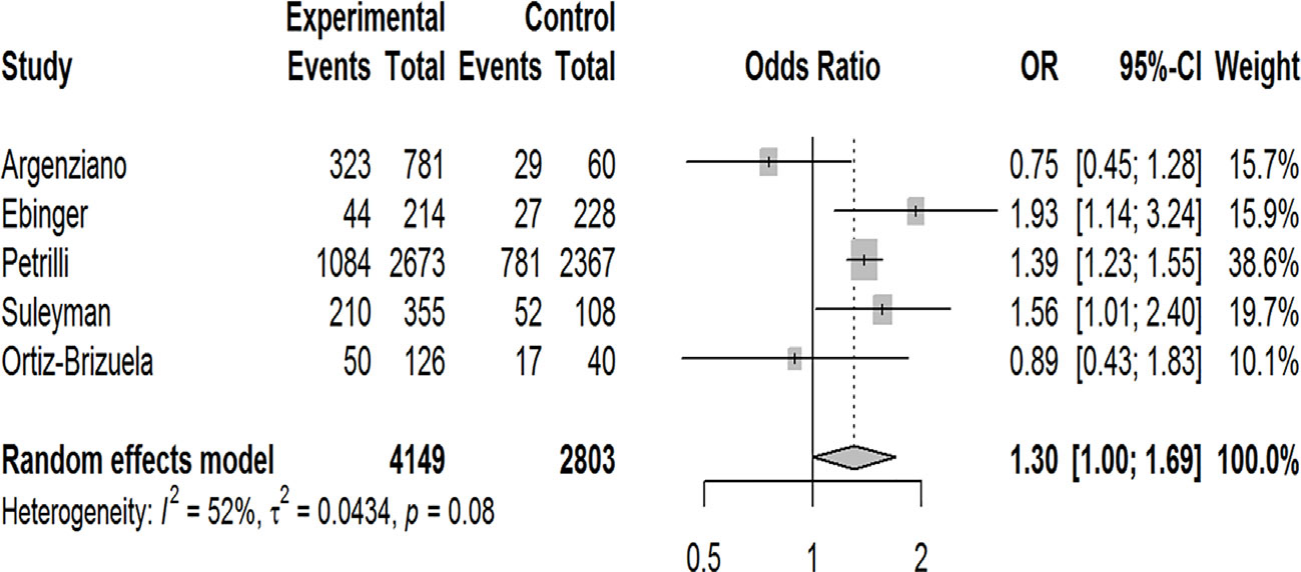
Forest-plot showing the association between obesity and hospitalization in patients with COVID-19.

**Figure 9:**
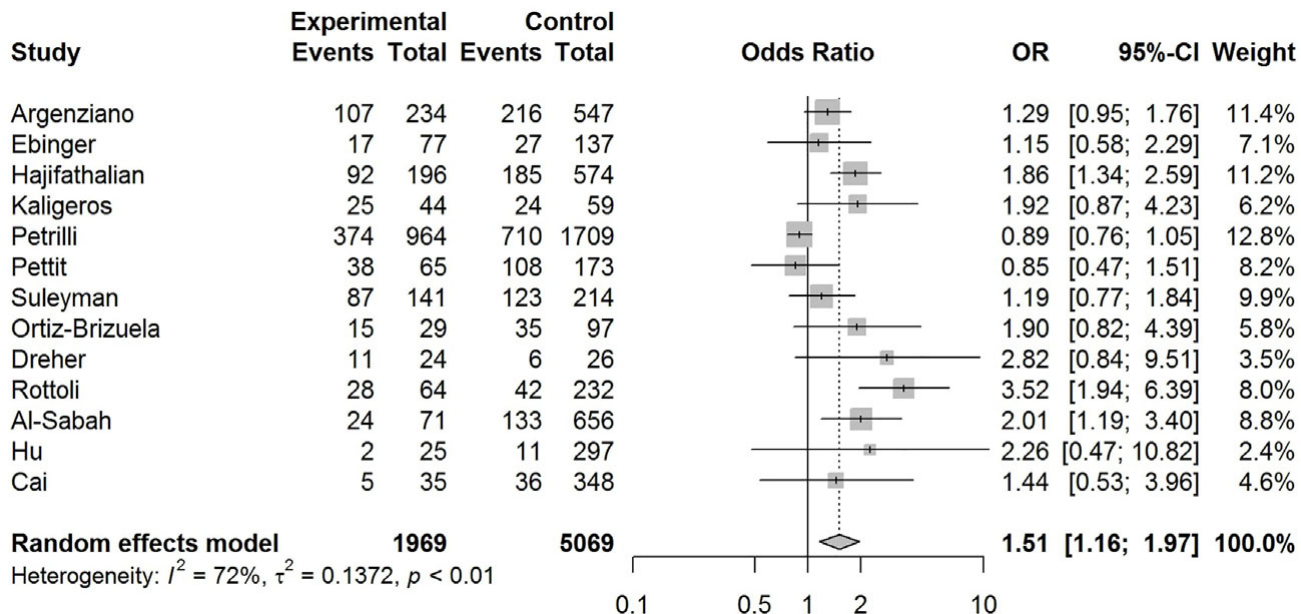
Forest-plot showing the association between obesity and admission to intensive care unit (ICU) in patients with COVID-19.

**Figure 10:**
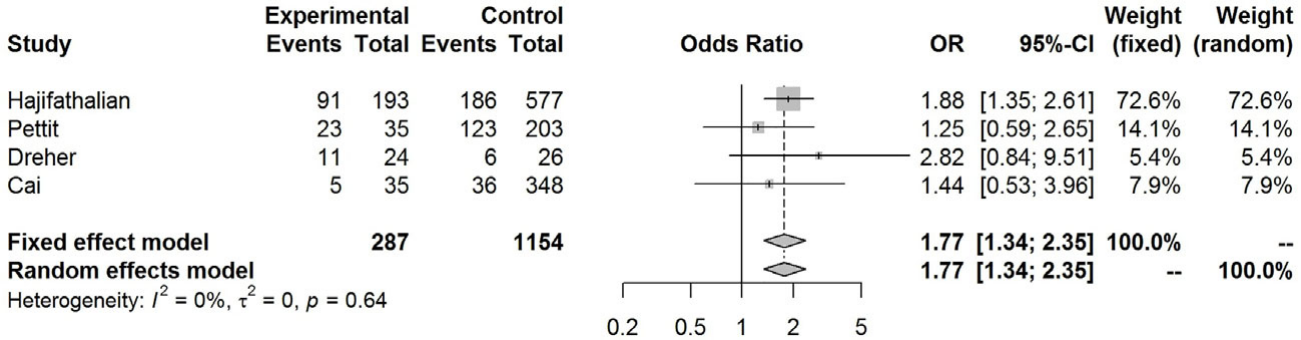
Forest-plot showing the association between obesity and invasive mechanical ventilation (IMV) requirement in patients with COVID-19.

**Figure 11:**
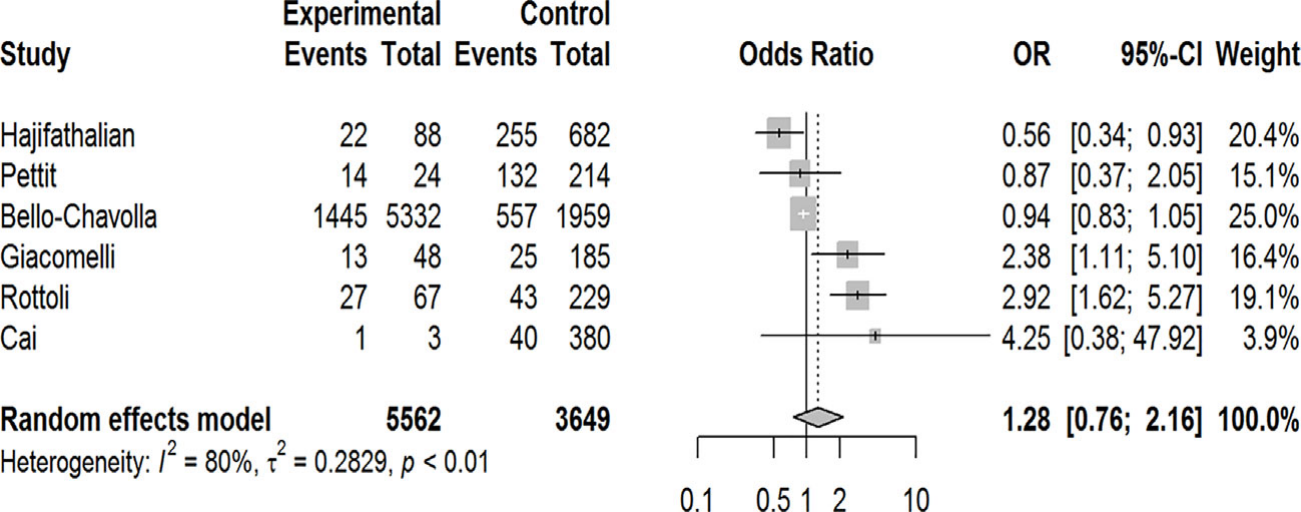
Forest-plot showing the association between obesity and death in patients with COVID-19.

Our pooled analyses showed that COVID-19 patients with obesity had a borderline higher risk for hospitalization [Odds ratio (OR):1.3, 95% CI: 1.00–1.69, p = 0.05; I^2^ 52%, p_heterogeneity_ = 0.08] (**Figure 8**). Obesity was related to significantly higher risk for ICU admission (OR:1.51, 95% CI: 1.16–1.97, p = 0.002; I^2^ 72%, p_heterogeneity_ < 0.01) (**Figure 9**), and IMV requirement (OR:1.77, 95% CI: 1.34–2.35, p < 0.001; I^2^ 0%, p_heterogeneity_ = 0.64) (**Figure 10**). However, obesity was not associated with increased risk for death in patients with COVID-19 (OR:1.28, 95% CI: 0.76–2.16, p = 0.35; I^2^ 80%, p_heterogeneity_ < 0.01) (**Figure 11**).

### Publication Bias

**Figure 12:**
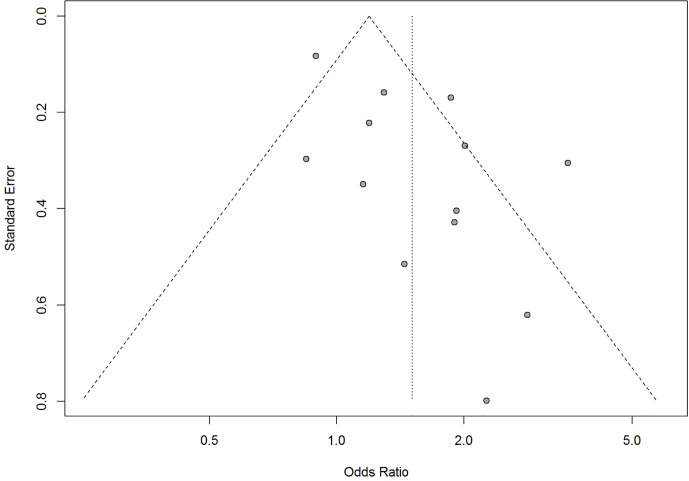
Funnel plot analysis for obesity and admission to intensive care unit (ICU).

The funnel-plot analysis for the association between obesity and risk of admission to ICU showed an asymmetrical shape (**Figure 12**), indicating the possibility of publication bias. Funnel-plot analysis was not performed for obesity and risk of hospitalization, IMV, and death because the number of included studies were less than 10.

## Discussion

In this systematic review and meta-analysis, we found that the pooled obesity prevalence rates were higher in patients with COVID-19 who were hospitalized, admitted to an ICU, or in need for IMV. Our pooled analysis revealed 1.3 times increased risk for hospitalization, 1.5 times increased risk for ICU admission, and approximately 1.8 times increased risk for IMV requirement among patients with obesity compared to patients without obesity.

In the fast-moving field of COVID-19, other meta-analyses have also assessed the relationship between this disorder and obesity. Using various inclusion criteria and definitions for the outcome variables, most of these meta-analyses indicated that in patients with COVID-19, obesity is significantly related to increased risk of severe disease and composite poor outcomes with reported ORs varying from 1.39 to 2.35 (33–41). A few meta-analyses presenting separate analyses for different outcomes reported increased risk of hospitalization with ORs varying from 1.4 to 2.13 (34, 42, 43), ICU admission with ORs varying from 1.21 to 1.74 (34, 43, 44), and IMV requirement with ORs varying from 1.66 to 2.29 (34, 36, 42–44) in obese patients with COVID-19. Our findings are in line with these reports in that we found 1.3 times higher risk for hospitalization, 1.51 times higher risk for ICU admission, and 1.77 times higher risk for IMV in obese patients with COVID-19 suggesting that obesity increases the severity of this disorder.

Prevalence of obesity varies across geographic region and obesity prevalence observed in hospitalized patients or in ICUs may depend on the local prevalence of obesity. Thus, we performed subgroup analysis by geographic region and found that in European countries, obesity was more common in patients with COVID-19 who needed ICU admission, IMV, or in those who died compared to background population. On the other hand, obesity rates only in COVID-19 patients requiring IMV exceeded the background general population prevalence of obesity in the U.S. In addition to the differences of obesity in the general population, the inconsistencies between Europe and the U.S. regarding the measured outcomes may be related with missing data, confounding factors, and variations in reporting methods and management protocols.

In our study, pooled prevalence rates of obesity seem to increase progressively with increasing disease severity, being highest in patients with IMV requirement. However, pooled obesity prevalence rate in patients who died was lower than that of those admitted to ICU or those with IMV requirement. Similar results were obtained in subgroup analysis by geographic region in the U.S. and Europe. Accordingly, our results showed that risk of death due to COVID-19 was not significantly increased in patients with obesity (OR:1.28, 95% CI: 0.76–2.16, p = 0.35). Consistent with our results, another meta-analysis including 34 studies from 9 different countries reported that obesity was significantly related to the increased risk of IMV during ICU admission, but not associated with excess mortality (OR: 1.15, 95% CI: 0.98–1.34) (36). These observations may be in agreement with obesity survival paradox. That is, despite the increased risk of pneumonia, pneumonia mortality might be lower in overweight and moderate obese individuals compared to those with normal BMI, as shown in a previous meta-analysis (45). Several hypothetical explanations have been proposed for this inverse association between obesity and risk of mortality, including clinicians’ lower threshold for ICU admission of obese patients, confounding, reverse causality, secretion of immunomodulatory substances by adipocytes (e.g., leptin, interleukin-10, and soluble TNF-α receptor) that may attenuate the inflammatory response, and the increased metabolic reserve provided by excess fat stores and lean body mass in obese patients that may counteract the increased catabolic stress and improve survival during critical illness (46). However, during the 2009 H1N1 pandemic, severe obesity (BMI ≥40 kg/m^2^) was identified as an independent risk factor for admission to ICU and death (47). Similarly, obesity has been reported to be associated with increased mortality due to COVID-19 in some of the very recently published meta-analyses with ORs varying from 1.37 to 3.68 (34, 35, 42, 43, 48) challenging the obesity survival paradox in COVID-19 in contrast to our results. The differences in the study populations and the discrepancies in the cut off values for BMI to define obesity among the included studies as well as the differences between healthcare systems, testing strategies, and indications for hospitalization, ICU admission, or IMV might explain these inconsistencies.

In the present study, we found that the pooled prevalence of obesity in all cases (admitted + non-admitted) with COVID-19 was 34% (22–46%). Among five studies that included both inpatient and outpatient confirmed COVID-19 cases, four were conducted in the U.S (9–12). and one in Mexico (19). Compared with the obesity prevalence in the general population of the U.S., this result suggests that obesity may not be associated with a higher test positivity for COVID-19. This contradicts the result of a study conducted in the United Kingdom (UK) (49). In this study, Yates et al., used UK Biobank data, in which 882 of 2,494 tests were positive for COVID-19. Although limited by possible selection bias, authors reported that both BMI and waist circumference were associated with testing positive for COVID-19 in a dose-response fashion. After adjustment for possible confounders, the OR for overweight, obese, and severe obese subjects was 1.31, 1.55, and 1.57, respectively, compared to those with healthy weight (49).

**Figure 13:**
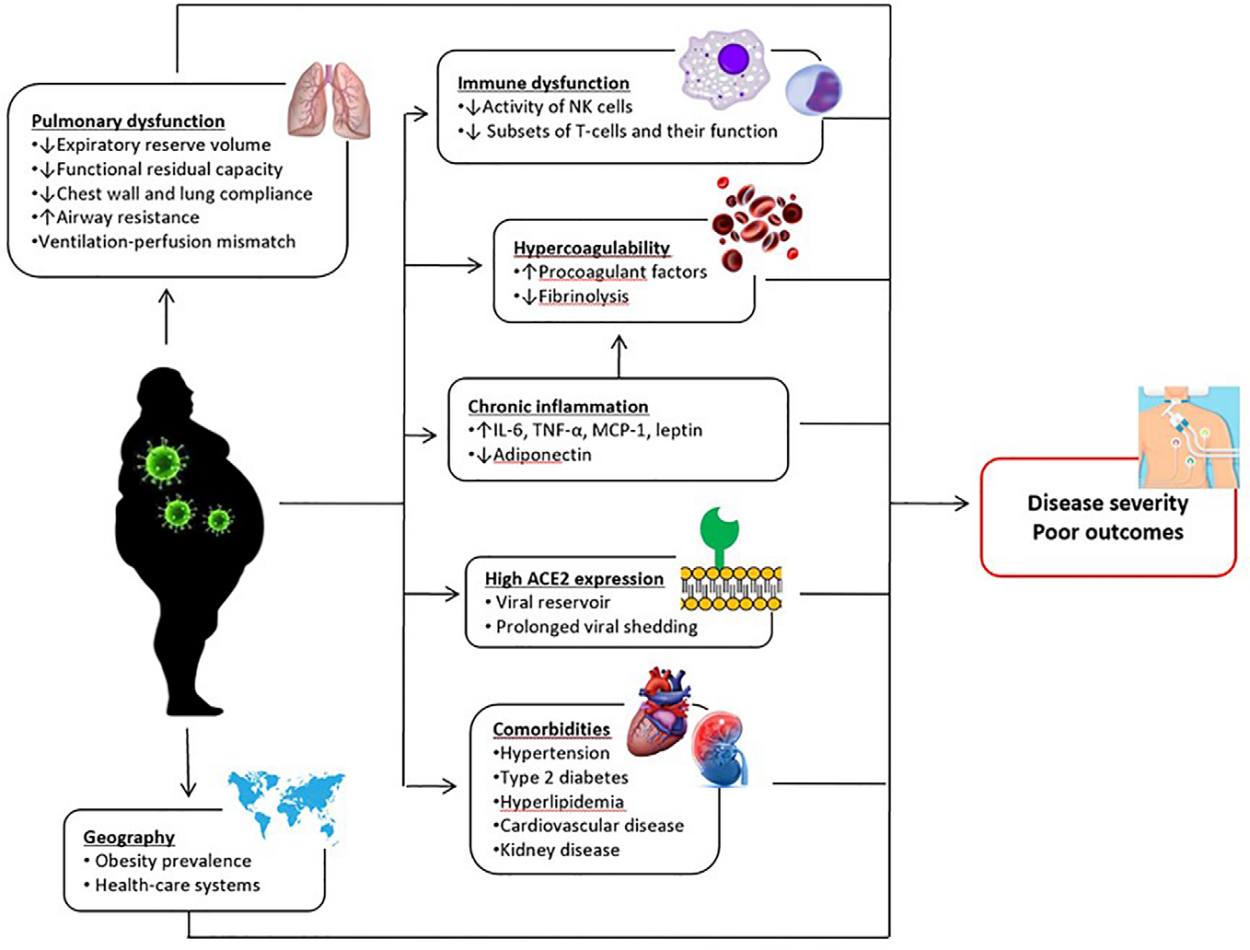
Postulated mechanisms underlying the relationship between obesity and poor COVID-19 outcomes. IL-6, interleukin-6; MCP-1, monocyte chemoattractant protein-1; NK, natural killer; TNF-α, tumor necrosis factor alpha.

Overall, our data indicate that obese subjects may be at higher risk for serious illness if infected and obesity may play a role in the progression of COVID-19. Several mechanisms have been proposed about the association between obesity and poor COVID-19 outcomes (**Figure 13**). Higher expression of angiotensin-converting enzyme 2 (ACE-2; an important functional receptor for SARS-CoV-2 invasion) in adipose tissue may lead to prolonged viral shedding and exposure in patients with obesity, increasing the susceptibility to SARS-CoV-2 infection and the risk of disease aggravation (35). Obesity driven chronic inflammation, aberrant cytokine activation, decreased adiponectin and increased leptin secretions, and dysfunction of innate and adaptive immunity may contribute to worse clinical outcomes in patients with COVID-19 (50–52). Obesity is also associated with hypercoagulability and increased risk of thrombosis (53), which seems to be one of the important factors leading to a more severe clinical course in infected patients. Moreover, obesity, particularly when it is severe, is associated with significant changes in pulmonary mechanics and respiratory muscle performance, which predispose patients to develop respiratory failure in the case of lung infection (54). Although these are plausible mechanisms, future studies are needed to prove that they are actually linked to COVID-19 outcomes.

This study, with its large sample size and inclusion of studies from different regions worldwide, presents further evidence about the relationship between obesity and COVID-19 outcomes. However, several limitations exist in this work. There was a significant heterogeneity among studies, probably due to the differences in sample sizes and baseline characteristics of the patients. Most included studies were retrospective and analyzed data of patients who were hospitalized, leading to a selection bias towards those with a more severe disease. Sampling and testing strategies, indications for hospitalization or admission to ICU, or indications for IMV were not adequately defined in most reports. Moreover, studies included in this analysis were short-term observational studies; outcomes could be different with a longer time of observation. Anthropometric measurements were not performed in most studies; using data from previous medical records may have led to incorrect BMI assessment and categorization. Besides, a substantial proportion of patients had missing BMI data in some studies. Furthermore, confounding factors including age, sex, ethnicity, deprivation, and comorbidities were not addressed in some reports. Given that obesity is related to these factors, it is difficult to interpret the potential role of obesity as an independent risk factor for poor COVID-19 outcomes. It should also be kept in mind that variations in treatment protocols over time across studies may have affected reported outcomes. Lastly, due to the small number of studies in subgroup analyses, strong conclusions could not be drawn on geographic variation of observations regarding the association between obesity and outcomes of COVID-19.

Despite its limitations, available data and results of our study consistently suggest that people living with obesity are at increased risk of poor COVID-19 outcomes. Thus, measurement of anthropometric parameters should be routinely performed in patients tested positive for COVID-19 as a part of risk assessment. Obese patients with COVID-19 should be followed and treated as a higher risk population. Testing priority, close monitoring, and earlier intensive treatment may be considered in these patients to avoid unfavorable clinical outcomes.

Special attention to obesity is also important in the aspect of disease prevention. Restriction on leaving home for several weeks was introduced in many countries as a measure to reduce rapid transmission of COVID-19. This may result in physical inactivity and, in long term, increase the susceptibility of people to develop obesity (55). Eventually, this may increase the number of individuals who will likely have a more severe course when infected with COVID-19. Thus, people should be encouraged to increase physical activity and gain healthy eating habits during pandemic.

In conclusion, our systematic review and meta-analysis indicated that the prevalence of obesity is higher in patients with severe COVID-19 and obesity is associated with increased risk for hospitalization, ICU admission, and IMV. However, abovementioned limitations of the included studies should be kept in mind while interpreting the results. Prospective cohort studies with a large sample size and addressing all potential confounding factors including age, sex, ethnicity, deprivation, and comorbidities are needed to clarify the independent role of obesity on the risk of COVID-19 and its clinical course. The pathogenesis of COVID-19 in patients with obesity should also be investigated to identify the causal mechanisms and interfere with prophylactic and therapeutic measures.

## Data Availability Statement

The original contributions presented in the study are included in the article/supplementary material. Further inquiries can be directed to the corresponding author.

## Author Contributions

BY supervised the project. BY and NE conceptualized the meta-analysis protocol. NH, NE, and BY screened the literature search results and assessed for the eligibility criteria. EK quantitatively synthesized the results of involved studies. NH and NE produced original form of the manuscript. BY reviewed and edited the manuscript. All authors contributed to the article and approved the submitted version.

## Funding

The authors received no financial support for the research and authorship of this article.

## Conflict of Interest

The authors declare that the research was conducted in the absence of any commercial or financial relationships that could be construed as a potential conflict of interest.
